# Current Challenges in Head and Neck Cancer Management

**DOI:** 10.3390/cancers14020358

**Published:** 2022-01-12

**Authors:** Anna Starzyńska, Bartosz Kamil Sobocki, Daniela Alterio

**Affiliations:** 1 Department of Oral Surgery, Medical University of Gdańsk, 80-211 Gdańsk, Poland; 2 Scientific Circle of Oncology and Radiotherapy, Medical University of Gdańsk, 80-211 Gdańsk, Poland; b.sobocki@gumed.edu.pl; 3 Scientific Circle of Oral Surgery, Medical University of Gdańsk, 80-211 Gdańsk, Poland; 4 Division of Radiotherapy, IEO European Institute of Oncology, IRCCS, 20141 Milan, Italy; daniela.alterio@ieo.it

**Keywords:** head and neck, cancer, treatment, diagnosis, prediction, prognosis, biomarkers

More than 500,000 new cases of head and neck cancer (HNC) occur each year worldwide [1]. HNC is mainly localized in the oral cavity, pharynx, larynx, salivary glands, and sinonasal cavities [2]. Although squamous cell carcinoma is the predominant histology, the final clinical outcome of HNC depends on many factors [1]. One of them is localization, which may be associated with different prognoses and levels of invasiveness [3,4]. A study concerning oral squamous cell carcinoma (OSCC) showed that different regions have specific molecular and histopathological signatures, which makes the TNM classification limited [3]. The major risk factors for squamous HNC are alcohol overuse, tobacco smoking, and human papillomavirus (HPV) infections. In the United States and western Europe, the smoking-related incidence of has HNC decreased, whereas HPV-associated incidence has increased [5]. Cases of HNC located in the oropharynx and hypopharynx region may be more strongly affected by alcohol than those located in the oral cavity and larynx [1]. The importance of localization in HNC outcome indicates the need for it to be appropriately reported in studies that concern HNC.

The early diagnosis of HNC remains a crucial factor in determining the final outcome of a patient due to limited therapeutic options in advanced and recurrent malignancies [6]. However, still there is a lack of efficient screening methods [7]. Even during standard pathological diagnosis, pathologists face challenges in their daily clinical routine. The discovery of new diagnostic biomarkers may be helpful in cases of poorly differentiated cancer or after adjuvant radiotherapy when it is hard to confirm cancer presence or recurrence [8]. Another aspect of HNC diagnosis is the use of non-invasive methods. There are some simple, but quite efficient methods such as exhaled breath analysis which still need clinical validation and evaluation [7]. Moreover, the status of liquid biopsy in HNC is still debatable and is a hot topic in the field. The analysis of circulating tumor DNA, intact circulating tumor cells, or exosomes may reveal metastasis earlier and help in monitoring response to therapy or residual disease post-treatment. The discovery of alterations in blood might have a potential predictive and prognostic role [9,10,11,12]. The analysis of p16-negative OSCC showed that 5-year disease-free survival rates decreased from 52 % in a group with zero positive lymph nodes to 21 % in a population of patients with three to four positive lymph nodes [13]. With the knowledge that early detection is crucial, new efficient biomarkers and methods should be investigated to improve the outcomes of HNC patients.

The modern treatment of HNC consists of surgery, radiotherapy, and systemic therapy [14]. Although many advancements in the treatment of cancer patients have been made, the 5-year survival rate of HNC patients has not been relevantly improved in recent decades [15]. This is the reason why we still need new targets for treatment. Currently, the rising role of immunotherapy can be observed in cancer. Additionally, pembrolizumab with platinum and fluorouracil have been approved by the FDA for patients with recurrent/metastatic disease and the positive expression of PD-L1 in the tumor [16]. In the case of platinum-resistant patients, the phase III Checkmate0141 study evaluated the efficacy of Nivolumab [17]. The results of new clinical trials which assess the effectiveness of T-Cell Inducible Co-Stimulatory Receptor Agonist and monalizumab, alone or in combination, are awaited [17]. Radiotherapy seems to be a promising direction, even in groups of patients with advanced or metastatic disease [18,19]. 

Clinical and radiological biomarkers of both the response to treatment and the toxicity profile of radiation therapy are currently very rare and are not routinely used in clinical practice for head and neck tumors. The “omics” approach represents one of the most intriguing fields of research. It could provide a large amount of information that could be used to predict radiation treatment response as well as the toxicity of patients [20]. Similarly, the tumor genomic profile seems to be a very promising tool for use in individualizing radiotherapy doses according to tumor radiosensitivity [21]. Moreover, the immune system status of patients (particularly the plasmatic neutrophil–lymphocyte ratio) has been demonstrated to be associated with both oncologic outcomes and radiation-related side effects [22,23]. These and other fields of investigation need to be developed to offer an even more personalized radiation treatment approach.

Finding new diagnostic, prognostic, and predictive biomarkers and promising targets for treatment may improve the prognosis of HNC patients. We would like to encourage researchers and scientists in this field to make a contribution to this Special Issue.
